# Supplementary figures and images for: Metabolite profiling of endophytic Streptomyces spp. and its antiplasmodial potential
Source: PeerJ. 2021 Mar 15;9:e10816. doi: 10.7717/peerj.10816 (PMC7971094; doi:10.7717/peerj.10816)

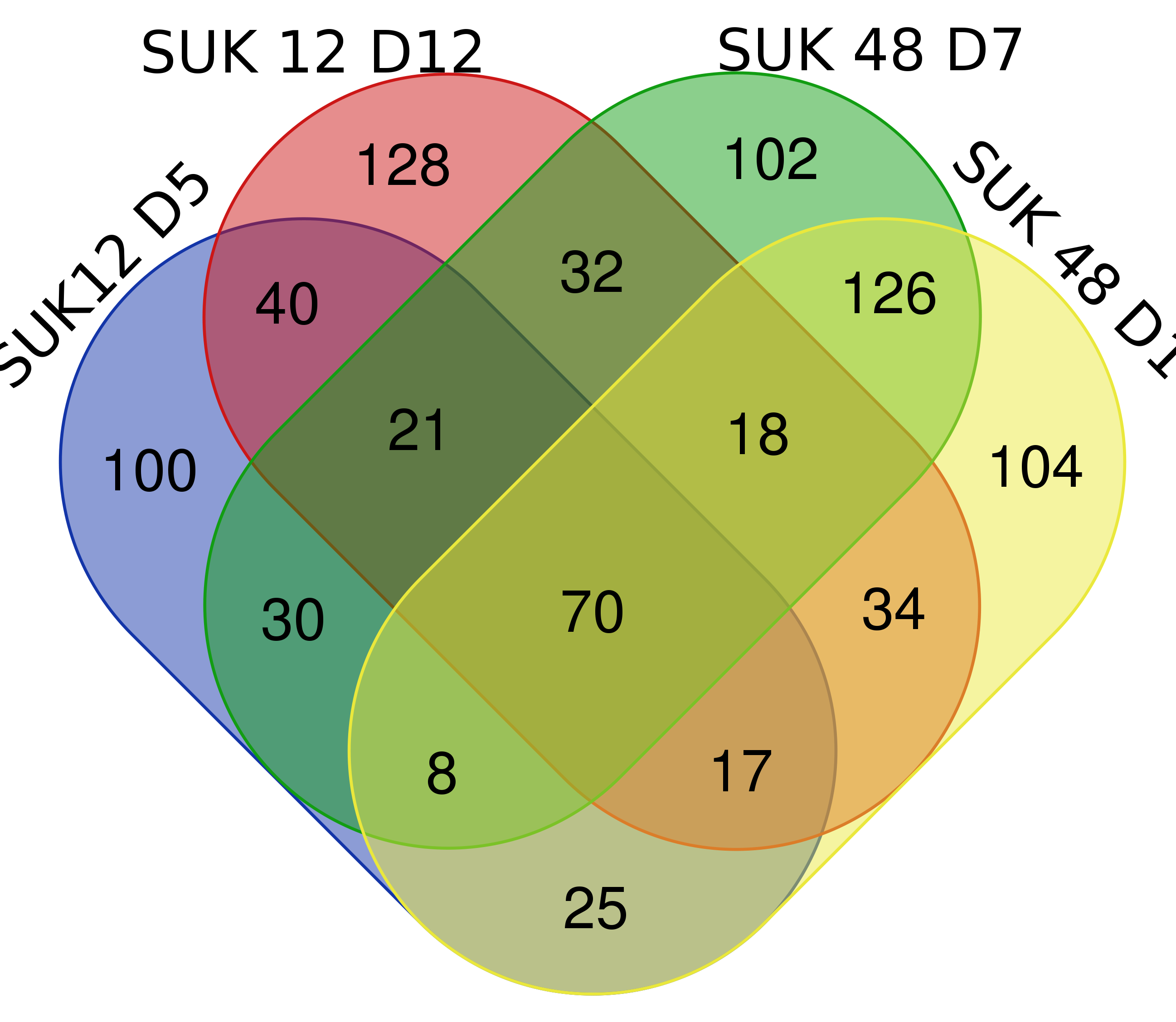

Supplement: Supplemental Information 4 — Venn Diagram of Streptomyces spp. metabolites. Commom metabolites of both Streptomyces are 70 metabolites. [file peerj-09-10816-s004.png]

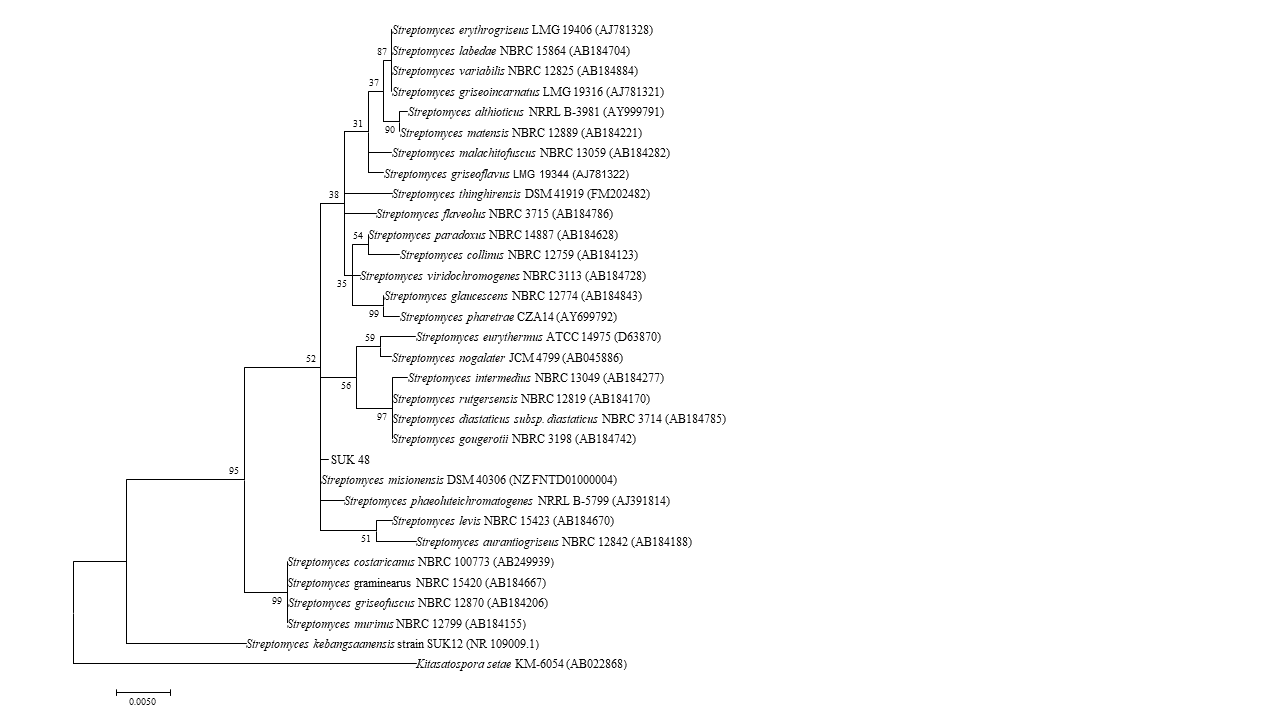

Supplement: Supplemental Information 6 — Phylogenetic tree of full length 16S rRNA nucleotide sequences using Maximum Likelihood method showing the relationship of strain SUK12 and SUK 48 with closely related members of the genus Streptomyces and Kitasatospora setae KM-6054T as the outgroup. Numbers at the nodes indicate levels of bootstrap support based on 1,000 replication. Bar, 0.005 changes per nucleotide. [file peerj-09-10816-s006.png]
